# Herpes Simplex Virus 1 Infection on a Reconstructive Free Flap

**Published:** 2013-06-04

**Authors:** Simon P. Parys, Thea Leman, Reuven Gurfinkel

**Affiliations:** Department of Plastic and Reconstructive Surgery, Sir Charles Gairdner Hospital, Perth, Australia

## Abstract

**Objective:** Herpes simplex virus 1 (HSV1) is a widespread virus that primarily causes orofacial infection. **Methods:** We present a case of HSV1 infection on a free radial forearm flap used to reconstruct a palate defect. Initially, the free flap appeared healthy; however, after 48 hours the free flap appeared in distress, with dark red colour and fast capillary refill. Venous congestion was suspected, and the patient underwent a second operation where no vascular compromise was found. Vesicles were noted on the free flap; swabs revealed HSV1 infection. **Results:** Complete recovery of the free flap was achieved with acyclovir. **Discussion:** To the best of our knowledge, this is the first report of HSV1 infection on a free flap that was found to be responsible for the free flap appearing distressed.

## OBJECTIVES

Herpes simplex virus (HSV1) can cause a significant oral infection, with clinical features including erythema and edema in association with transient white vesicles. In the case presented, this clinical picture was supported by laboratory-confirmed HSV1. We postulate that the HSV1 infection caused the congested appearance of the free flap. This necessitated an urgent reexploration, where no vascular compromise was found.

We believe that this is the first reported case of HSV1 infection on a free flap as well as HSV1 being responsible for the distressed appearance of a free flap.

## METHODS

**Figure 1:**
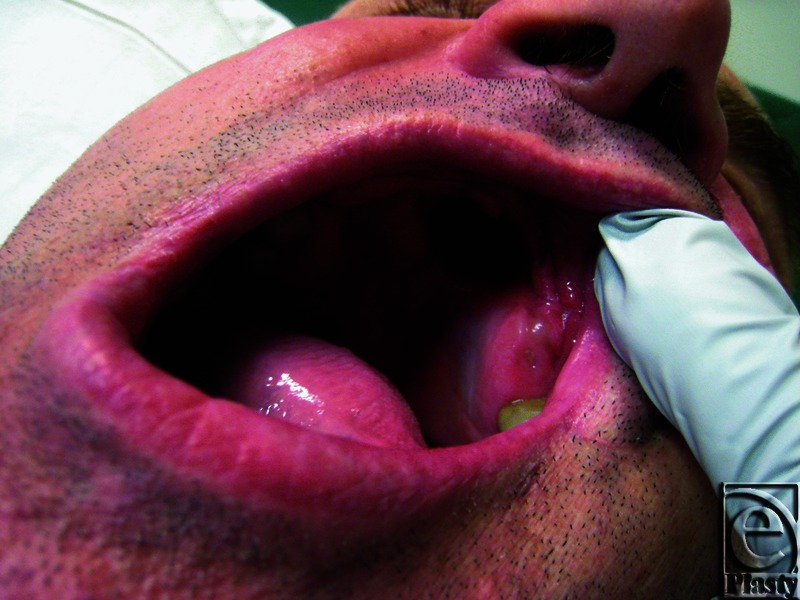
Hard palate defect before surgery.

A 40-year-old Caucasian man was referred to the plastic surgery department for the repair of a hard palate defect (Fig 1). The defect was the result of a self-inflicted gunshot wound 15 years ago.

The patient was an ex–intravenous drug user with a strong dependence on opiates.

Oral mucosa from the edge of the defect was used as a turnover flap to create the nasal lining. A free radial forearm flap was harvested from the left forearm to create the oral mucosa to repair the palate defect with anastomosis performed to the left facial artery and vein. Ischemic time was 1 hour 38 minutes. Drain and nasogastric tubes were inserted, and postoperatively the patient was placed in a heated room, head up at 30° with half-hourly flap observations.

For the first 48 hours, the postoperative recovery was unremarkable, with the free flap being pink, soft, and warm with a strong audible Doppler signal. Small erythematous patches began to appear at the lateral edge of the flap.

Over the next 24 hours, the free flap became firmer, more swollen, and bruised with a strong Doppler signal. The free flap appeared to be in distress with dark color and fast capillary refill, and venous congestion was suspected.

On day 3, the patient returned to the operating theatre for exploration of the vascular anastomosis. The arterial anastomosis had adequate flow and was not revised. The venous anastomosis was reexplored, but no clot or kink was found. An additional venous anastomosis was performed, with the previously raised cephalic vein being anastomosed to the external jugular vein. The anterior margin of the free flap was reopened and washed, after which the flap was reinset.

**Figure 2:**
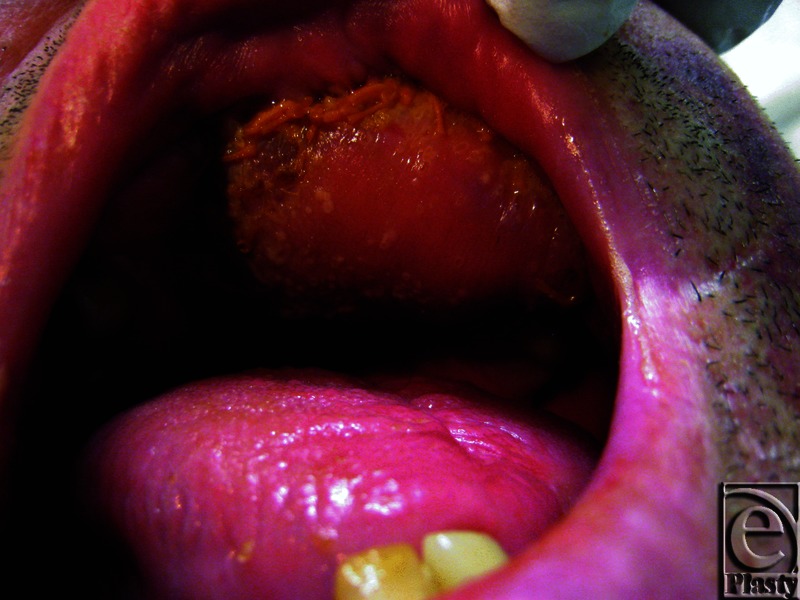
Free flap day 4. Note the white vesicles present on the free flap.

On day 4, the free flap appeared dark pink (Fig 2). Capillary refill was 1 second and a strong audible Doppler signal was present. White vesicles were noted on the flap. Microscopy and culture swabs were taken. An Infectious Disease and Dermatology review was sought. The possibility of *Candida* was raised and nystatin oral drops commenced.

**Figure 3:**
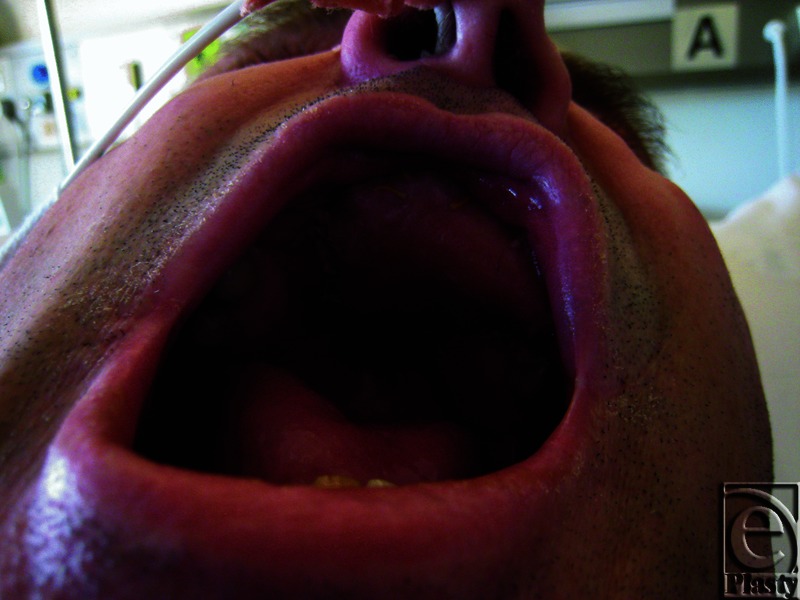
Flap after acyclovir treatment.

By day 7, the results of the previously taken swabs became available with polymerase chain reaction nucleic acid detection test positive for HSV1. The patient was commenced on acyclovir tablets, 400 mg 5 times daily, given via a nasogastric tube with a fast response and complete recovery of the free flap (Fig 3).

## RESULTS

After the treatment the free flap was warm and pink with brisk capillary refill and a strong audible Doppler signal. The surface was sloughing off. The patient was discharged home on day 15 and commenced on soft diet on day 18. The patient recovered well and long-term follow-up is ongoing.

## DISCUSSION

Herpes simplex virus 1 is one of more than 80 different strains of the herpes virus family, primarily affecting skin and mucous membranes.^1^ It is present, depending on the age, in between 60% and 90% of the adult population.^2^ The majority of primary HSV1 infections are subclinical and not recognized; however, infection can be associated with paresthesia, lymphadenopathy, and viral syndrome. Over several days numerous white vesicles develop, and the mucosa may become inflamed, erythematous, or even purple and edematous.^3^ Subsequently, HSV1 becomes latent but can reactivate, usually causing the common cold sore, but can present in a similar fashion to primary herpes.

Herpes simplex virus 1 is known to cause infection in surgical wounds, most commonly in burn wounds and postburn skin grafts.^4^^-^6 Excluding burns, there are only single reports of HSV1 infection at a knee arthroscopy port site,^7^ skin cancer excision site,^8^ and gingival graft.^9^

A review of postoperative infection in head and neck surgery revealed the expected causes of infection, namely bacteria, including *Staphylococcus aureus*, *Streptococcus*, *Pseudomonas*, Gram-negative bacteria, and anaerobes as well as fungal infections, most commonly *Candida*.^10^^-^14 Although viral reactivation after surgery is well known with various different viruses,^15^^,^^16^ there is, to the best of our knowledge, no previous report of an HSV1 infection on a free flap nor HSV1 infection resulting in a distressed appearance of a free flap. Although surgical site infections in head and neck reconstructions are not rare, partly because of contamination from oropharyngeal bacteria, these infections rarely necessitate an urgent reexploration.^17^^,^^18^

The reexploration rate in head and neck reconstructions varies between 3% and 19%, with clinical deterioration the primary indication.^17^^-^19 If vascular compromise is clinically suspected, an expedient reexploration is associated with improved rates of flap salvage.^18^ The most common findings at reexploration are venous thrombus, arterial thrombus, and hematoma. There is a small rate of negative reexplorations, that is, reexplorations where no vascular thrombosis or collection is found. These negative explorations are most commonly performed for clinical signs of venous congestion.^18^

In our case, at day 3, the free flap appeared congested with brisk capillary refill, swollen texture, darker colour, and strong Doppler signals. This prompted a reexploration with the working diagnosis of a venous occlusion. No venous thrombosis, occlusion, or kink was found. In the presented case, the clinical findings were not typical of venous congestion; however, the vesicles were apparent only on day 4 (after the reexploration), and HSV1 was only diagnosed at day 7, all of which made the differential diagnosis harder. It is our opinion to urgently reexplore the vascular anastomosis of any free flap that clinically appears in vascular distress.

Treatment of HSV1 includes oral preparations of antivirals such as acyclovir or valacyclovir as well as topical agents including acyclovir or penciclovir. Treatment, if started early, reduces the severity and duration of infection but has no effect on latent HSV.^1^
